# Knowledge, attitudes, and practices towards artificial intelligence among young pediatricians: A nationwide survey in France

**DOI:** 10.3389/fped.2022.1065957

**Published:** 2022-12-23

**Authors:** Emma Perrier, Mahmoud Rifai, Arnaud Terzic, Constance Dubois, Jérémie F. Cohen

**Affiliations:** ^1^ Child Neurological Rehabilitation Unit and Learning Disorders Reference Centre, Assistance Publique-Hôpitaux de Paris, Hôpital Bicêtre, Université Paris-Saclay, Le Kremlin-Bicêtre, France; ^2^ Pediatric Intensive Care Unit, Assistance Publique-Hôpitaux de Paris, Hôpital Raymond-Poincaré, Université Paris-Saclay, Paris, France; ^3^ Pediatric Intensive Care and Neonatal Medicine, Assistance Publique – Hôpitaux de Paris, Hôpital Bicêtre, Université Paris-Saclay, Le Kremlin-Bicêtre, France; ^4^ Centre of Research in Epidemiology and Statistics, Inserm UMR 1153, Université Paris Cité, Paris, France; ^5^ Department of General Pediatrics and Pediatric Infectious Disease, Assistance Publique – Hôpitaux de Paris, Hôpital Necker – Enfants Malades, Université Paris Cité, Paris, France

**Keywords:** artificial intelligence, pediatrics, knowledge - attitude - behavior, survey, machine learning

## Abstract

**Objective:**

To assess the knowledge, attitudes, and practices (KAP) towards artificial intelligence (AI) among young pediatricians in France.

**Methods:**

We invited young French pediatricians to participate in an online survey. Invitees were identified through various email listings and social media. We conducted a descriptive analysis and explored whether survey responses varied according to respondents’ previous training in AI and level of clinical experience (i.e., residents vs. experienced doctors).

**Results:**

In total, 165 French pediatricians participated in the study (median age 27 years, women 78%, residents 64%). While 90% of participants declared they understood the term “artificial intelligence”, only 40% understood the term “deep learning”. Most participants expected AI would lead to improvements in healthcare (e.g., better access to healthcare, 80%; diagnostic assistance, 71%), and 86% declared they would favor implementing AI tools in pediatrics. Fifty-nine percent of respondents declared seeing AI as a threat to medical data security and 35% as a threat to the ethical and human dimensions of medicine. Thirty-nine percent of respondents feared losing clinical skills because of AI, and 6% feared losing their job because of AI. Only 5% of respondents had received specific training in AI, while 87% considered implementing such programs would be necessary. Respondents who received training in AI had significantly better knowledge and a higher probability of having encountered AI tools in their medical practice (*p* < 0.05 for both). There was no statistically significant difference between residents’ and experienced doctors’ responses.

**Conclusion:**

In this survey, most young French pediatricians had favorable views toward AI, but a large proportion expressed concerns regarding the ethical, societal, and professional issues linked with the implementation of AI.

## Introduction

Artificial intelligence (AI) pioneer Marvin Minsky defined AI as “the science of making machines do things that would require intelligence if done by men” (Box 1) (1). The use of AI tools is rapidly increasing in clinical medicine, thanks to the generalized availability of powerful computers and large datasets (2). So far, AI has been mainly used to develop diagnostic tools for various medical conditions (3). For example, with image recognition techniques such as convolutional neural networks, AI may help clinicians detect fractures on x-rays (4), diabetic retinopathy on digital fundus images (5), skin cancer (6), and genetic diseases on facial images (7). Beyond convolutional neural networks, other AI-enabled diagnostic applications include, for instance, improvement in diagnosing autism (8), identification of child abuse from medical records (9), and natural language processing systems to assist clinicians with detecting rare conditions (10). AI also has many non-diagnostic applications, including support for managing chronic diseases such as diabetes (11), decision support and hospital monitoring systems, drug discovery pipelines, and surgical robots (12).

Box 1 **Definition of key terms used in artificial intelligence literature.**

• Artificial intelligence (AI): AI is a broad term that refers to the ability of a digital computer or computer-controlled robot to perform tasks commonly associated with human intelligence.
• Machine learning (ML): ML is a subfield of AI. ML is a method to train a computer to learn from its raw input to perform complex regression and classification tasks.
• Neural network (NN): A NN is an ML program that operates in a manner inspired by the organization of neurons in the human brain.
• Deep learning (DL): DL is a class of NN that  uses a high number of neuron-type units and layers to hierarchically extract features from the raw input. Popular applications of DL are in image and speech recognition.

AI-based healthcare technologies are promising, but they rely on complex statistical methods and concepts, thus generating high expectations but also fears among clinicians. Several studies have investigated the knowledge, attitudes, and practices (KAP) of young healthcare professionals towards AI. For example, Sit et al. conducted an online survey *via* social media among 484 medical students in the UK (13). This study aimed to identify their KAP towards AI and the potential impact of AI on choosing radiology as a specialization (13). While 49% of students stated that they were less willing to specialize in radiology out of fear of AI, 88% of participants believed AI would play an important role in medicine, and 89% claimed that AI training would benefit their career. Participants who had received theoretical training in AI were significantly more inclined to specialize in radiology and felt more confident using AI-based tools in their future practice.

In France, a qualitative survey conducted by Laï et al. focused on the perception of AI among various healthcare workers (14). Forty individuals were interviewed: 13 physicians, 7 individuals involved in the industry, 5 researchers in the field of AI, 7 members of regulatory agencies, and 8 people who were not directly involved in the development of AI but had previously written about the challenges of implementing AI in medicine. Healthcare professionals appeared focused on providing their patients with the best and safest care. The responses revealed that AI seemed a true breakthrough for healthcare industrial partners, but legal difficulties in accessing individual health data could hamper its development. Institutional players were aware of their significant role in regulating the use of AI tools. Healthcare researchers specializing in AI had a more pragmatic point of view and hoped for a better translation from research to practice.

Other studies focusing on medical AI surveyed radiologists (15, 16), psychiatrists (17), and dermatologists (18). However, to our knowledge, no study focused specifically on the KAP towards AI among young pediatricians. In this context, we invited young French pediatricians to participate in an online survey.

## Materials and methods

### Study design and participant selection

This is a cross-sectional online study using several regional (i.e., Ile de France, the Paris area) and national mailing lists and social media. We aimed at including young pediatricians registered on the Facebook groups “Internes de France” (*n* = 17,096), “Association des Juniors en Pédiatrie” (AJP; *n* = 1,267), “Promo 2019 de Pédiatrie” (*n* = 87), “Pédiatrie Paris Promo socle” (*n* = 88), “Pédiatrie Paris Promo 2020” (*n* = 93) as well as on AJP-Paris’ mailing lists, regrouping Ile-de-France pediatrics residents from 2016 to 2021 (*n* = 465; Appendix 1). The survey was anonymous. Only pediatricians were eligible; young physicians from other specialties were excluded. There were no strict age limits, but most social media groups we used were targeted at residents and fellows. AJP's mailing list includes young pediatricians, mostly below 30 years old, all below 40. Data collection was conducted from January 12 to February 16, 2022. We followed the CHERRIES statement for reporting (Appendix 2).

### Survey tool

The online questionnaire was developed and administered *via* Google Form. Consent, age, sex, faculty of origin, level of experience, and any additional training were gathered from the initial seven questions. The survey was then structured into four parts: (1) knowledge about AI (5 questions), (2) expected benefits of AI (13 questions), (3) fears toward AI (8 questions), and (4) practices regarding AI (6 questions). Responses to closed questions were collected on a 5-point Likert scale (i.e., “totally disagree”, “rather disagree”, “neutral”, “rather agree”, “totally agree”). Answers to questions expecting a numerical entry were offered a range of plausible values. Each social media and mailing list received a separate questionnaire, and the response files were merged for analysis. There was no need to answer all the questions of the survey to be included in the study, and partial responses were kept in the analysis. Because questionnaires were anonymous, it was not possible to detect and exclude duplicates, but we believe it is very unlikely that respondents took the survey several times.

### Data categorization

The “residents” category included residents from the first to the eighth semester of residency training. The “experienced doctors” category included chief residents (“Docteur Junior” status), physicians working under a resident contract (“Faisant Fonction d’Interne” status), fellows (“Assistant/Chef de Clinique” status), attending physicians, professors (assistant, associate, and full), and private practitioners. We considered the responses “totally agree” and “rather agree” as positive, and the responses “rather disagree” and “totally disagree” as negative. Neutral responses were considered a third response category.

### Statistical analysis

We first performed a descriptive analysis of study participants and survey responses. Descriptive statistics included means and medians for qualitative variables. Survey responses were summarized as percentages. In an exploratory approach, we used Chi-square tests and Fisher's exact tests (if *n* < 5) to compare proportions and assess if responses varied according to whether respondents had received (specific or non-specific) training in AI. We also compared residents’ and experienced doctors’ responses. All analyses involved the use of R software (R Foundation, Austria, Vienna). The significance threshold was set at 0.05. There was no specific sample size calculation for this survey.

### Ethics

Participation in the survey was voluntary. A short paragraph was included at the beginning of the questionnaire to inform participants of the study's objectives and of the confidentiality of their responses. Consent was considered obtained by virtue of questionnaire completion. Data were collected anonymously, and participants had the right to access and cancel their answers. In accordance with French legal regulations, ethical approval was not required for this study.

## Results

### Participant characteristics

**Figure 1:**
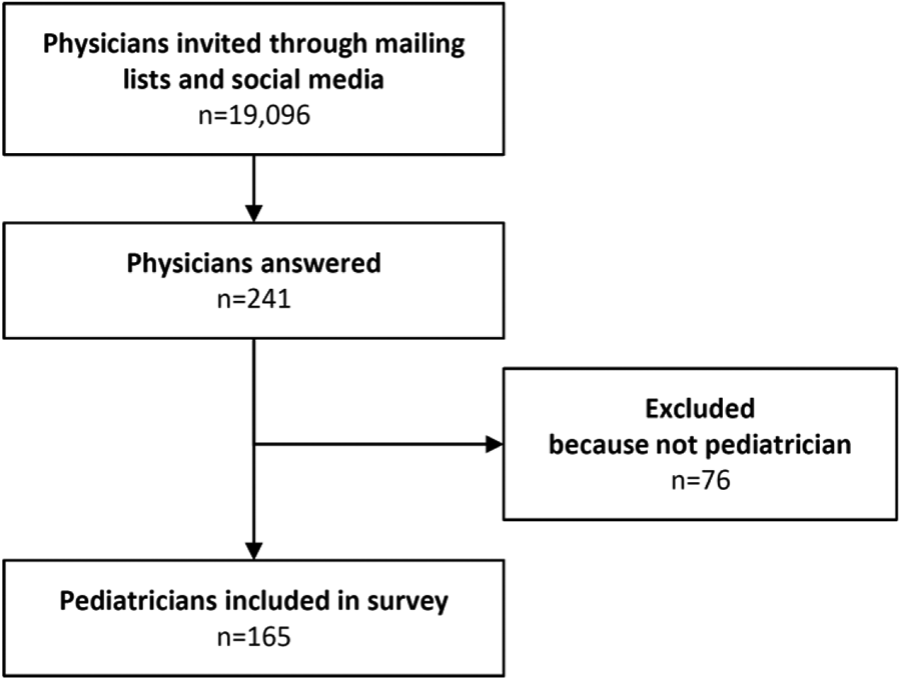
Flowchart.

One hundred and sixty-five pediatricians responded to the survey (Figure 1) The participation rate was difficult to estimate due to potential redundancies across social media groups and mailing lists. Respondents’ median age was 27 years (interquartile range 25–30 years), and 78% of respondents were women. In total, 75% of the participants attended medical school in Ile-de-France (Paris V, Paris VI, Paris VII, UPEC, Paris-Sud, Paris 13, Versailles-Saint-Quentin Universities). Regarding clinical experience, 64% of respondents were classified as residents, while 36% of included pediatricians were “experienced doctors”.

### Knowledge about AI

In total, 90% of the participants stated that they understood the term “artificial intelligence”, compared to 65% for “machine learning”, 54% for “neural network” and only 40% for “deep learning”. Only 31% of the participants reported that they knew the difference between these different terms, while half of them (49%) did not. From a regulatory perspective, 58% of the respondents declared they were familiar with the General Data Protection Regulation (GDPR) framework.

### Attitudes toward AI

The majority of the surveyed pediatricians seemed to have a favorable view of AI (Table 1). The participants appeared to think that AI could lead to major improvements in medicine, particularly in terms of medical training (88%), better access to healthcare in settings where experts are not available (80%), diagnostic assistance (71%), better compliance with treatment and patient follow-up (91%), and help for choosing among various therapeutic options (73%). More than half (60%) of respondents agreed with the statement that AI would “revolutionize medicine” while a quarter (24%) preferred to stay neutral on this question. A majority (86%) declared that they would favor the implementation of AI tools in pediatrics.

**Table 1:** Survey responses: expected benefits of AI.

Question	*n*/*N*	%
Do you think AI will improve medical training?		
Yes	145/164	88
No	7/164	4
Neutral	12/164	7
Do you think AI will facilitate information gathering from patients?		
Yes	117/165	71
No	23/165	14
Neutral	25/165	15
Do you think AI will help analyze patient medical data to come up with potential diagnoses?		
Yes	122/165	74
No	18/165	11
Neutral	25/165	15
Do you think AI will improve healthcare access, more particularly where experts are not available?		
Yes	132/164	80
No	14/164	9
Neutral	19/164	11
Do you think AI will improve patient compliance with treatment and follow-up?		
Yes	150/165	91
No	7/165	4
Neutral	8/165	5
Do you think AI will help analyze patient medical data to assess prognosis?		
Yes	101/165	61
No	35/165	21
Neutral	29/165	18
Do you think AI will help analyze patient medical data to offer the most appropriate therapeutic options?		
Yes	120/164	73
No	19/164	12
Neutral	25/164	15
Do you think AI will revolutionize medicine?		
Yes	99/164	60
No	25/164	15
Neutral	40/164	24
Are you in favor of implementing AI tools in your specialty?		
Yes	142/165	86
No	3/165	2
Neutral	20/165	12

More than half of respondents (59%) saw AI as a threat to medical data security and 35% as a threat to the ethical and human dimensions of medicine (Table 2). Regarding their practice of medicine, 39% feared skill loss induced by task delegation to AI, but only 6% of the pediatricians stated that they feared losing their job because of AI.

**Table 2:** Survey responses: fears toward AI.

Question	*n*/*N*	%
Are you afraid that it would be challenging to create a legal regulatory framework for AI use in healthcare?		
Yes	103/165	62
No	34/165	21
Neutral	28/165	17
Are you afraid that AI may threaten patient data security?		
Yes	98/165	59
No	49/165	30
Neutral	18/165	11
Do you see AI as a threat to the ethical and human dimensions of medicine?		
Yes	58/165	35
No	77/165	47
Neutral	30/165	18
Are you afraid to lose your job because of AI?		
Yes	10/165	6
No	136/165	82
Neutral	19/165	12
Are you afraid doctors will lose some of their skills if AI is implemented in their workflow?		
Yes	98/165	59
No	46/165	28
Neutral	21/165	13

### Practices regarding AI

Only 5% of the pediatricians stated that they had received specific teaching in AI, and 29% of pediatricians declared having been in contact with AI through specific or non-specific training (Table 3). In total, 42% of the participants declared that they had encountered at least one AI tool in their clinical practice. The vast majority of respondents considered that it would be necessary to implement specific training and courses about AI (87%).

**Table 3:** Survey responses: practices regarding AI.

Question	*n*/*N*	%
Were you ever offered specific training in AI during medical school?		
Yes	8/164	5
No	156/164	95
Have you ever encountered AI tools in your theoretical training?		
Yes	119/165	72
No	46/165	28
Have you ever been in contact with AI through specific or non-specific training?		
Yes	48/164	29
No	116/164	71
Have you ever encountered AI tools in your medical practice?		
Yes	69/165	42
No	96/165	58
Do you think that doctors should receive specific training on the use of AI tools in healthcare?		
Yes	143/165	87
No	2/165	1
Neutral	20/165	12
Do you think that doctors should receive specific training regarding the societal and ethical challenges of AI in healthcare?		
Yes	154/165	93
No	2/165	1
Neutral	9/165	5

### Exploratory association analyses

Forty-eight physicians (29%) declared they had contact with AI through (specific or non-specific) training. These respondents significantly more often stated: (i) to know the difference between the terms “artificial intelligence”, “machine learning”, “neural network”, and “deep learning” (44% vs. 26%, *p* = 0.027), (ii) to know what the General Data Protection Regulation (GDPR) is (73% vs. 51%, *p* = 0.009), and (iii) to have encountered AI tools in their medical practice (65% vs. 33%, *p* < 0.001). We found no statistically significant difference between residents’ and experienced doctors’ responses (Appendix 3).

## Discussion

### Main findings

In this nationwide online survey of 165 young French pediatricians, we assessed their KAP towards AI in healthcare. We found insufficient knowledge in the lexical field and core concepts of AI, as, for example, 49% of the participants did not know the differences between the terms “artificial intelligence”, “machine learning”, “neural network” and “deep learning”. We also observed that the majority of respondents seemed to have a positive view of AI and were in favor of implementing AI tools in pediatrics. In contrast, more than half of respondents saw AI as a threat to medical data security and one-third as a threat to the ethical and human dimensions of medicine. Forty-two percent of the participants declared that they had encountered at least one AI tool in their medical practice, whereas only 5% of the pediatricians stated that they had received specific teaching about AI in medical school. Respondents who received training in AI had significantly better knowledge and a higher probability of encountering AI tools in their medical practice (Appendix 3).

### Comparison with previous literature

To our knowledge, this is the first study focusing on the KAP towards AI among pediatricians in France. Similar studies have been conducted in other fields of medicine, including radiology (15, 16), psychiatry (17), and dermatology (18), but not in pediatrics, while several AI applications are being implemented in this specialty (3, 12).

Our finding that less than a third (31%) of pediatricians knew the difference between “artificial intelligence”, “machine learning”, “neural network”, and “deep learning” is in line with other studies that also questioned physicians on their understanding of AI [response rates: 24% among dermatologists (18) and 35% among radiologists (16)]. Only 5% of the pediatricians in our survey said they had received specific training in AI, but 42% had encountered AI solutions in their practice. In the study by Ooi et al. among radiologists (16), a similar proportion of only 5% of respondents stated that they had received training in AI. Still, the majority of them commonly used AI solutions in their practice (93% for voice recognition and 56% for image interpretation) (16). It appears that specific training on AI in medical studies is currently lacking. Hence, doctors may confront AI tools directly in their clinical practice without prior instruction about the concepts behind algorithms, how AI solutions are developed and evaluated, and their limitations and potential biases (19, 20). Accordingly, 87% of the pediatricians in our survey considered that it would be necessary to offer specific training in AI, as 80% of the dermatologists surveyed by Polesie et al. (18) and 89% of the radiologists in the Ooi study (16).

Regarding their attitudes toward AI in medicine, 60% of young French pediatricians believed that AI would “revolutionize medicine”. A greater proportion (89%) of radiologists surveyed in the Ooi study agreed that AI would “revolutionize the practice of their specialty” (16). This higher proportion could be because the vast majority (93%) of radiologists already commonly used AI solutions in their practice (16). In addition, radiology relies on advanced technologies, whereas pediatricians might be more focused on clinical skills. In the study surveying dermatologists (18), 69% of respondents believed that AI would “revolutionize dermatology”. However, the authors explained that “dermatologists with a special interest in dermatoscopy were more likely to have received the invitation [to participate in the survey]”, potentially shifting the results toward a positive perception of AI.

Regarding the potential negative attitudes toward AI, only 6% of pediatricians expressed their fear of losing their job because of AI. This low proportion was also found in dermatology (5.4%) (18). Also, in psychiatry, only 3.8% of responding physicians feared that AI would make their practice obsolete, but as high as 75% of them thought that AI could replace them in completing and updating medical records (17). Coppola's study among 1,032 radiologists reported that 11% of them were afraid of being replaced by an AI solution (15). This shows that most physicians across several specialties see AI as having the potential to help clinicians rather than replace them.

Attitudes towards AI were generally positive: 86% of French pediatricians in our survey favored implementing AI tools in their specialty, as were 77% of radiologists in Coppola's study (15). When asked whether increased use of AI would make their specialty more “exciting”, 63% of dermatologists (18) and 76% of radiologists agreed (16). AI thus seems generally perceived as a solution to enhance clinical practice.

### Limitations

Our study has limitations. First, there was probably a selection bias due to our distribution channels favoring access to young pediatricians trained in the Paris region (75%). This population of young pediatricians might be more exposed to research and innovation, including AI, in their training and clinical practice than in other regions. Furthermore, determining the exact response rate was not practicable because of the potential for user overlap between groups. Given that in 2022, France counted 1,635 residents training in Pediatrics, our best approximation of the response rate in this subgroup is around 6% (105/1,635). Hence, our collected responses may not represent the KAP of all young French pediatricians. Second, we opted for a relatively short and shallow questionnaire to maximize the completion rate. Qualitative and mixed-methods studies are needed to allow deeper investigations of barriers and facilitators of AI use in pediatrics.

## Conclusion

While AI offers many promises in healthcare, it raises technical, professional, and ethical questions. The majority of young French pediatricians who responded to this survey had positive attitudes towards AI and emphasized the need to set up specific training programs in AI and the importance of ethical and societal issues linked with the implementation of AI in healthcare.

## Data Availability

The raw data supporting the conclusions of this article will be made available by the authors, without undue reservation.

## Appendix 1 Mailing lists and social media used to invite participants.

**Table:**

Name	Type	National/Regional	Target	*N*
Internes de France	Facebook group	National	Residents (all specialties)	17,096
Association des Juniors en Pédiatrie	Website	National	Young pediatricians	1,267
Promo 2019 de Pédiatrie	Facebook group	Regional (Paris region)	Residents (pediatrics)	87
Pédiatrie Paris Promo socle	Facebook group	Regional (Paris region)	Residents (pediatrics)	88
Pédiatrie Paris Promo 2020	Facebook group	Regional (Paris region)	Residents (pediatrics)	93
AJP-Paris	Mailing list	Regional (Paris region)	Young pediatricians	465

## Appendix 2 Checklist for Reporting Results of Internet E-Surveys (CHERRIES).

**Table:**

Checklist item	Explanation	Page number
Describe survey design	Describe target population, sample frame. Is the sample a convenience sample? (In “open” surveys this is most likely.)	5
IRB approval	Mention whether the study has been approved by an IRB.	7
Informed consent	Describe the informed consent process. Where were the participants told the length of time of the survey, which data were stored and where and for how long, who the investigator was, and the purpose of the study?	7
Data protection	If any personal information was collected or stored, describe what mechanisms were used to protect unauthorized access.	No personal information collected
Development and testing	State how the survey was developed, including whether the usability and technical functionality of the electronic questionnaire had been tested before fielding the questionnaire.	5–6
Open survey versus closed survey	An “open survey” is a survey open for each visitor of a site, while a closed survey is only open to a sample which the investigator knows (password-protected survey).	5
Contact mode	Indicate whether or not the initial contact with the potential participants was made on the Internet. (Investigators may also send out questionnaires by mail and allow for Web-based data entry.)	5
Advertising the survey	How/where was the survey announced or advertised? Some examples are offline media (newspapers), or online (mailing lists – If yes, which ones?) or banner ads (Where were these banner ads posted and what did they look like?). It is important to know the wording of the announcement as it will heavily influence who chooses to participate. Ideally the survey announcement should be published as an appendix.	5
Web/E-mail	State the type of e-survey (eg, one posted on a Web site, or one sent out through e-mail). If it is an e-mail survey, were the responses entered manually into a database, or was there an automatic method for capturing responses?	5–6
Context	Describe the Web site (for mailing list/newsgroup) in which the survey was posted. What is the Web site about, who is visiting it, what are visitors normally looking for? Discuss to what degree the content of the Web site could pre-select the sample or influence the results. For example, a survey about vaccination on a anti-immunization Web site will have different results from a Web survey conducted on a government Web site	Appendix 2
Mandatory/voluntary	Was it a mandatory survey to be filled in by every visitor who wanted to enter the Web site, or was it a voluntary survey?	5–6
Incentives	Were any incentives offered (eg, monetary, prizes, or non-monetary incentives such as an offer to provide the survey results)?	No incentive
Time/Date	In what timeframe were the data collected?	5
Randomization of items or questionnaires	To prevent biases items can be randomized or alternated.	No randomization
Adaptive questioning	Use adaptive questioning (certain items, or only conditionally displayed based on responses to other items) to reduce number and complexity of the questions.	No adaptive questioning
Number of Items	What was the number of questionnaire items per page? The number of items is an important factor for the completion rate.	5
Number of screens (pages)	Over how many pages was the questionnaire distributed? The number of items is an important factor for the completion rate.	5
Completeness check	It is technically possible to do consistency or completeness checks before the questionnaire is submitted. Was this done, and if “yes”, how (usually JAVAScript)? An alternative is to check for completeness after the questionnaire has been submitted (and highlight mandatory items). If this has been done, it should be reported. All items should provide a non-response option such as “not applicable” or “rather not say”, and selection of one response option should be enforced.	6
Review step	State whether respondents were able to review and change their answers (eg, through a Back button or a Review step which displays a summary of the responses and asks the respondents if they are correct).	7
Unique site visitor	If you provide view rates or participation rates, you need to define how you determined a unique visitor. There are different techniques available, based on IP addresses or cookies or both.	Undetermined
View rate (Ratio of unique survey visitors/unique site visitors)	Requires counting unique visitors to the first page of the survey, divided by the number of unique site visitors (not page views!). It is not unusual to have view rates of less than 0.1% if the survey is voluntary.	Undetermined
Participation rate (Ratio of unique visitors who agreed to participate/unique first survey page visitors)	Count the unique number of people who filled in the first survey page (or agreed to participate, for example by checking a checkbox), divided by visitors who visit the first page of the survey (or the informed consents page, if present). This can also be called “recruitment” rate.	Undetermined
Completion rate (Ratio of users who finished the survey/users who agreed to participate)	The number of people submitting the last questionnaire page, divided by the number of people who agreed to participate (or submitted the first survey page). This is only relevant if there is a separate “informed consent” page or if the survey goes over several pages. This is a measure for attrition. Note that “completion” can involve leaving questionnaire items blank. This is not a measure for how completely questionnaires were filled in. (If you need a measure for this, use the word “completeness rate”.)	100%
Cookies used	Indicate whether cookies were used to assign a unique user identifier to each client computer. If so, mention the page on which the cookie was set and read, and how long the cookie was valid. Were duplicate entries avoided by preventing users access to the survey twice; or were duplicate database entries having the same user ID eliminated before analysis? In the latter case, which entries were kept for analysis (eg, the first entry or the most recent)?	No cookies
IP check	Indicate whether the IP address of the client computer was used to identify potential duplicate entries from the same user. If so, mention the period of time for which no two entries from the same IP address were allowed (eg, 24 h). Were duplicate entries avoided by preventing users with the same IP address access to the survey twice; or were duplicate database entries having the same IP address within a given period of time eliminated before analysis? If the latter, which entries were kept for analysis (eg, the first entry or the most recent)?	No IP check
Log file analysis	Indicate whether other techniques to analyze the log file for identification of multiple entries were used. If so, please describe.	No log file analysis
Registration	In “closed” (non-open) surveys, users need to login first and it is easier to prevent duplicate entries from the same user. Describe how this was done. For example, was the survey never displayed a second time once the user had filled it in, or was the username stored together with the survey results and later eliminated? If the latter, which entries were kept for analysis (eg, the first entry or the most recent)?	Not aplicable
Handling of incomplete questionnaires	Were only completed questionnaires analyzed? Were questionnaires which terminated early (where, for example, users did not go through all questionnaire pages) also analyzed?	6
Questionnaires submitted with an atypical timestamp	Some investigators may measure the time people needed to fill in a questionnaire and exclude questionnaires that were submitted too soon. Specify the timeframe that was used as a cut-off point, and describe how this point was determined.	Undetermined
Statistical correction	Indicate whether any methods such as weighting of items or propensity scores have been used to adjust for the non-representative sample; if so, please describe the methods.	No statistical correction performed

This checklist has been modified from Eysenbach G. Improving the quality of Web surveys: the Checklist for Reporting Results of Internet E-Surveys (CHERRIES). J Med Internet Res. 2004 Sep 29;6(3):e34 [erratum in J Med Internet Res. 2012; 14(1): e8.]. Article available at https://www.jmir.org/2004/3/e34/; erratum available https://www.jmir.org/2012/1/e8/. Copyright ©Gunther Eysenbach. Originally published in the Journal of Medical Internet Research, 29.9.2004 and 04.01.2012. This is an open-access article distributed under the terms of the Creative Commons Attribution License (https://creativecommons.org/licenses/by/2.0/), which permits unrestricted use, distribution, and reproduction in any medium, provided the original work, first published in the Journal of Medical Internet Research, is properly cited.

## Appendix 3 Exploratory association analyses

a. Responses according to whether physicians declared they had contact with AI through (specific or non-specific) training.

**Table:** 1. Do you know the difference between the terms “artificial intelligence”, “machine learning”, “neural network”, and “deep learning”?

Answer	Had contact with AI through (specific or non-specific) training		Total
	Yes	No	
Yes	21	30	51
No or Neutral	27	85	112
Total	48	115	163

44% vs. 26%, *p* = 0.027.

**Table:** 2. Do you know what the General Data Protection Regulation (GDPR) is?

Answer	Had contact with AI through (specific or non-specific) training		Total
	Yes	No	
Yes	35	59	94
No or Neutral	13	57	70
Total	48	116	164

73% vs. 51%, *p* = 0.009.

**Table:** 3. Have you encountered AI tools in your medical practice?

Answer	Had contact with AI through (specific or non-specific) training		Total
	Yes	No	
Yes	31	38	69
No or Neutral	17	78	95
Total	48	116	164

65% vs. 33%, *p* < 0.001.

b. Comparison of residents’ and experienced doctors’ responses.

**Table:** 1. Do you know the difference between the terms “artificial intelligence”, “machine learning”, “neural network”, and “deep learning”?

Answer	Respondent type		Total
	Resident	Experienced doctor	
Yes	32	19	51
No or Neutral	73	40	113
Total	105	59	164

30% vs. 32%, *p* = 0.819.

**Table:** 2. Do you know what the General Data Protection Regulation (GDPR) is?

Answer	Respondent type		Total
	Resident	Experienced doctor	
Yes	64	31	95
No or Neutral	41	29	70
Total	105	60	165

61% vs. 52%, *p* = 0.246.

**Table:** 3. Have you encountered AI tools in your medical practice?

Answer	Respondent type		Total
	Resident	Experienced doctor	
Yes	44	25	69
No or Neutral	61	35	96
Total	105	60	165

42% vs. 42%, *p* = 0.976.
